# The effect of the 7R allele at the DRD4 locus on risk tolerance is independent of background risk in Senegalese fishermen

**DOI:** 10.1038/s41598-022-27002-3

**Published:** 2023-01-12

**Authors:** Gwen-Jirō Clochard, Aby Mbengue, Clément Mettling, Birane Diouf, Charlotte Faurie, Omar Sene, Emilie Chancerel, Erwan Guichoux, Guillaume Hollard, Michel Raymond, Marc Willinger

**Affiliations:** 1 Ecole polytechnique - CREST (CNRS-UMR 9194), Palaiseau, France; 2 University of Chicago and Universidad del CEMA’s Joint Initiative for Latin American Experimental Economics, Chicago, USA; 3 Université Gaston Berger, Saint-Louis, Senegal; 4 CNRS, IRD, ISEM Univ Montpellier, Montpellier, France; 5 CNRS, IGH Univ Montpellier, Montpellier, France; 6 Université Alioune Diop, Bambey, Senegal; 7 INRAE, BIOGECO, Plateforme Genome Transcriptome de Bordeaux, Univ Bordeaux, 33610 Cestas, France; 8 CNRS, INRAE, CEE-M Univ Montpellier, Montpellier, France

**Keywords:** Heritable quantitative trait, Population genetics

## Abstract

It has been shown that living in risky environments, as well as having a risky occupation, can moderate risk-tolerance. Despite the involvement of dopamine in the expectation of reward described by neurobiologists, a GWAS study was not able to demonstrate a genetic contribution of genes involved in the dopaminergic pathway in risk attitudes and gene candidate studies gave contrasting results. We test the possibility that a genetic effect of the DRD4-7R allele in risk-taking behavior could be modulated by environmental factors. We show that the increase in risk-tolerance due to the 7R allele is independent of the environmental risk in two populations in Northern Senegal, one of which is exposed to a very high risk due to dangerous fishing.

## Introduction

Humans need to adapt their behavior as a result of risk. Previous research has shown that risk coping attitudes are partly heritable^1^. Genes involved in the regulation of the dopaminergic system are good candidates to explain the heritability of risk behavior. However, many reports on gene and behavior association, based on small-sample candidate gene have found contrasting results, leading to debates in the scientific community^2,3^.

To overcome this limitation, a genome-wide association study (GWAS), based on over 1 million individuals, identified 99 loci associated with general risk tolerance^4^. Surprisingly, none of identified loci were close to genes involved in the dopamine pathway. Their bioinformatic analysis pointed to the role of genes expressed in brain regions involved in decision-making, although genes involved in dopamine biosynthesis (TH) or receptors (DRD1,2,3 and 4) did not reach statistical significance.

Yet, the evidence that not only these brain regions but the dopamine neurotransmitter itself plays a role in the expectation of reward is compelling: dopaminergic neurons can code the probability of the reward in a primate model^5^. Moreover, a known side-effect of the treatment of Parkinson disease (known to impair dopamine production) is to dramatically increase impulsivity^6^. The dopamine receptor gene DRD4 fulfills many criteria as a good candidate gene: it is highly polymorphic^7,8^, expressed in the prefrontal cortex, it shows an unusually large variable repeat region (VNTR: variable number tandem repeat) coding for 16 amino acids in the third cytoplasmic loop, a region interacting with SH3 domain-binding proteins.

While the 4 repeat (4R) variant is the ancestral, and predominant allele in most human populations^9^, there exist variations between 2 and 11 repeats (2R to 11R). The different alleles have functional differences^10–13^. The DRD4-7R allele is under strong positive selection in human population^14–16^, and has been shown to be linked to more risk-tolerant attitudes^17–19^. However, some findings revealed a lack of differences in the domain of financial risk-taking^20–23^.

The discrepancy between these studies may come from the fact that GWAS studies tend to underestimate the genetic variance due to gene-gene or gene-environment interactions, or an inability to capture rare genetic variants. Furthermore, candidate-gene studies conducted in specific environments may sometimes benefit from circumstances revealing a genetic variance. For instance, administration of L-DOPA to volunteers did not lead to an increase in gambling propensity unless the subjects carried at least one copy of the 7-repeat allele^24^. The negative association between DRD4 variation and risk-taking previously reported^23^ might have been concealed by the association with MAOA variation, an enzyme catalysing dopamine. It is therefore likely that the effect of genetic variants of DRD4 on risk-taking behavior appears only in specific circumstances. GWAS studies, by leveling all environmental conditions or gene interactions, may mask some dopaminergic genetic contributions. Two studies have also shown the effect of DRD4 to be modulated by an interaction with maternal effects^25,26^.

Humans also adapt their risk attitudes as a response to the level of risk in their environment^27^. In particular, people have been found to be more risk-averse in the presence of unfair background risk^28–31^, in accordance with the “risk-vulnerability” hypothesis^32,33^.

The aim of the present paper is to test the interaction between the influence of the 7R allele on risk-tolerance and the level of risk to which people are exposed.

## Results

### Risk-tolerance by zone

The village of Guet Ndar (Saint-Louis region in Northern Senegal) is famous for its fisheries. Fishing in the area is very dangerous, with authorities reporting 20 deaths due to fishing on average per year over the past 20 years^34^. Given the demography of the village, with 20 000 inhabitants, among which fishing represents the main occupation of approximately 80% of the adult male workforce, this corresponds to approximately 4% of the male population who died due to fishing in the last 20 years. The prevalence of deaths is strongly linked to the intersection of strong currents coming from the Senegal river and an upwelling current from the ocean^35^. However, these currents attract a lot of fish, making fishing more profitable than other activities in the region (fishermen in our sample declare income significantly higher than non-fishermen, $p < 0.01$, Table S.1).

In this paper, we compared populations from the fishing village of Guet Ndar (*N = 601*), which is labelled as the *risky area*, and that of a farming village called Mouit, 23 kilometers away (*N = 263*), labelled the *non-risky area*. Importantly, the two populations are mostly composed of the same ethnic group (the Wolofs, representing approximately 80% of the sample in both areas). Because fishing is an activity predominately performed by men, our sample only consists of men. Only participants over 18 years old were allowed to participate. No further restrictions on participants were placed, e.g. based on residency, activity. Descriptive statistics are provided in Table S.2.

**Figure 1:**
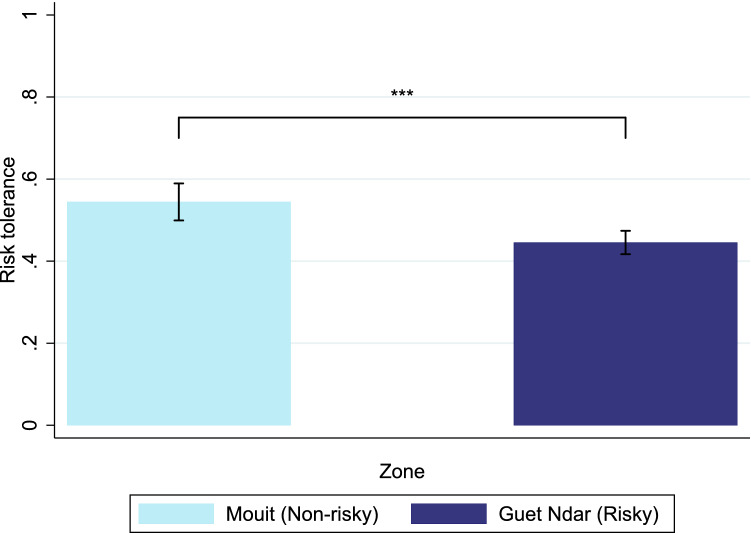
Average level of risk-tolerance by zone. Note: This figure shows the average level of risk-tolerance between the risky and non-risky areas. A higher level of risk-tolerance indicates the choice of a riskier lottery by participants in the lottery choice task. Segments represent 95% confidence intervals. Student’s *t*-test * $p<$ 0.1, ** $p<$ 0.05, *** $p<$ 0.01.

Our experimental measure of risk-tolerance was based on a lottery task^36^. A description of the task is provided in the Supplementary Materials. Results indicate that risk-tolerance varied between the risky and non-risky areas. Participants from the risky area tended to exhibit less risk-tolerance than participants from the non-risky area (Figure 1, Student’s *t*-test $p < 0.01$). The difference remains significant after controlling for age and education (Table S.3). Our data is consistent with field data and laboratory experiments showing that people exposed to high background risk tend to exhibit less risk-tolerance, in accordance with the “risk-vulnerability hypothesis”^32,33^.

### Genotypes by zone

Genotypes at the DRD4 locus displayed two common alleles (4R and 7R, with 4 and 7 repeats, respectively), which was expected for populations in Sub-Saharan Africa^9^, and 5 minor alleles with negligible frequencies (2R, 3R, 5R, 6R and 8R) leading to 21 different genotypes (Table 1). The 7R allele was more prevalent than previously found in other African populations^9^, possibly because the ethnic groups from participants were not sampled in previous work. In Chang et al., 1996, the groups analyzed are Bantu and San Bushmen, from South-Africa and Namibia, Biaka from the Central African Republic, Mbuti from the Democratic Republic of Congo and Falasha in Ethiopia. The closest group from our sample geographically would be the Biaka people from the Central African Republic, 5,000 km away.

Within each area, populations were not at Hardy-Weinberg equilibrium ($p<0.01$ in the non-risky area, $p=0.02$ in the risky area), and displayed an heterozygote deficiency ($F_{IS}$ = 0.134 in the non-risky area, and $F_{IS}$ = 0.052 in the risky area).

Because we were primarily interested in the effect of the 7R allele on risk-taking, we combined all other alleles into a single category, identified as allele “X”. This combination yielded three genotypes: XR/XR, XR/7R and 7R/7R. Hardy-Weinberg equilibrium was rejected ($p = 0.01$) for the non-risky area, but not for the risky area ($p = 0.40$), see Table 1. Deviations from HW equilibrium were $F_{IS}$ = 0.181 in the non-risky area, and $F_{IS}$ = 0.037 in the risky area.

We found evidence of limited migration. In the risky area, 81% of our sample were born in the same village, as were 72% of their parents, and 68% of grand parents. Numbers were slightly lower for the non-risky area, with 67% of participants, 58% of their parents and 50% of their grand parents (Table S.6).

The genotypic differentiation between the two areas was measured as $F_{ST}$ = 0.0036, and was marginally non-significant (exact G test, *p* = 0.094). This level of genotypic differentiation was compared with those displayed by 30 micro-satellite loci. One locus (032) was not polymorphic and was discarded. The other 29 loci displayed between 2 and 15 alleles. Their level of genotypic differentiation ranged between $F_{ST}$ = -0.0094 and $F_{ST}$ = 0.0226, with an overall average value of $F_{ST}$ = 0.0035 (Figure S.2 and Table S.8).

**Table 1:** Genotypic composition at the *DRD4* locus of populations from the Saint-Louis region in the non-risky and risky areas.

Genotype	Non-risky area		Risky area	
	*N*	%	*N*	%
P**anel A. Without combination of genotypes**				
22	3	1.4	3	0.6
24	7	3.3	14	2.8
25	1	0.5	–	–
27	–	–	2	0.4
34	3	1.4	–	–
36	1	0.5	–	–
37	1	0.5	–	–
44	84	39.1	202	40
45	19	8.8	34	6.7
46	14	6.5	18	3.6
47	48	22.3	149	29.5
48	5	2.3	12	2.4
55	4	1.9	3	0.6
56	–	–	1	0.2
57	4	1.9	14	2.6
58	–	–	2	0.4
66	2	0.9	1	0.2
67	1	0.5	12	2.4
77	15	7	34	6.7
78	3	1.4	4	0.8
88	–	–	1	0.2
**HW equilibrium**				
*p*	<0.01		0.023	
**Panel B. Allele 7R versus other alleles**				
XX	143	66.5	291	57.5
X7	57	26.5	181	35.8
77	15	7.0	34	6.7
**HW equilibrium**				
*p*	0.011		0.40	

The *p*-value (*p*) corresponds to the HW probability exact test. Genotype ij refers to the *DRD4* genotype iR/jR. For *Panel B*, all alleles not 7R are combined in the X allele.

### Risk-tolerance by genotype

**Figure 2:**
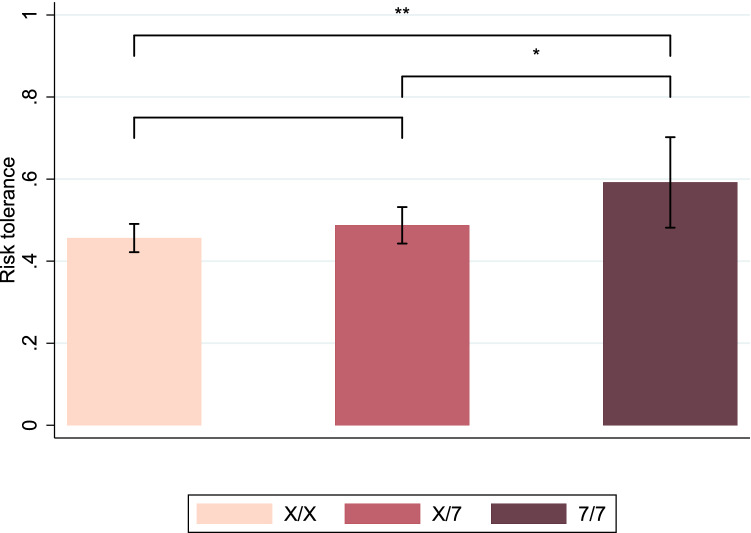
Average level of risk-tolerance by genotype. Note: This figure shows the average level of risk-tolerance between the different genotypes. A higher level of risk-tolerance indicates the choice of a riskier lottery by participants in the lottery choice task. X/X, X/7 and 7/7 represent genotypes, with all alleles not 7R combined into the X allele. Samples from both risky and non-risky areas are pooled. Segments represent 95% confidence intervals. Student’s *t*-test * $p<$ 0.1, **$p<$ 0.05.

Risk-tolerance was not independent of genotype at the DRD4 locus (Figure 2 and Table 2, Column 1). The 7R allele demonstrated a significant additive effect ($p=0.01$), and no dominance effect was found ($p=0.31$). The 7R allele increased risk-tolerance. Importantly, the result held after controlling for age, education and the living area (Table 2, Column 2). Our results indicated that the 7R allele was associated with more risk-tolerance than other alleles, in line with previous literature^17,18^. Moreover, we did not find that the 7R allele was associated with a measure of novelty-seeking (Table S.5).

Environmental risk did not appear to significantly moderate the effect of the 7R allele. First, the additive effect held when analyzing both areas separately (Table 2, Columns 3 and 4, Figure S.1), although the significance levels dropped slightly due to sample limitations ($p = 0.05$ and $p = 0.08$ in the non-risky and risky area, respectively). Second, the interaction between the additive effect and the area (Table 2, Column 5) was not significant ($p = 0.25$).

**Table 2:** Differences between genotypes in risk-tolerance.

	(1)	(2)	(3)	(4)	(5)
	Combined	Combined	Non-risky	Risky	Gene-
	sample	sample	area	area	environment
	without	with	only	only	interaction
	controls	controls			
7R: additive effect	0.068$$^{**}$$	0.064$$^{**}$$	0.097$$^{*}$$	0.056$$^{*}$$	0.107$$^{**}$$
	(0.027)	(0.028)	(0.049)	(0.032)	(0.045)
7R: dominance effect	−0.037	−0.026	−0.003	−0.036	−0.024
	(0.036)	(0.037)	(0.069)	(0.042)	(0.037)
Age		−0.002$$^{*}$$			−0.002$$^{**}$$
		(0.001)			(0.001)
Education		−0.004			−0.004
		(0.005)			(0.005)
Risky area		−0.120$$^{***}$$			−0.094$$^{**}$$
		(0.032)			(0.038)
Risky area $\times$ 7R: additive effect					−0.062
					(0.049)
Constant	0.456$$^{***}$$	0.613$$^{***}$$	0.507$$^{***}$$	0.431$$^{***}$$	0.597$$^{***}$$
	(0.017)	(0.053)	(0.030)	(0.021)	(0.054)
Observations	721	699	215	506	699
R$^{2}$	0.009	0.030	0.026	0.006	0.033

*Note:* The outcome variable is risk-tolerance. A higher level of risk-tolerance indicates the choice of a riskier lottery by participants in the lottery choice task. Standard errors in parentheses. The coefficients are the results of Ordinary Least Square (OLS) estimations. In column 1, the sample is pooled (non-risky and risky areas) and no control variable is included. In column 2, the sample is pooled and controls for age, education and a dummy for living in the risky area are included. In columns 3 and 4, the sample is restricted to individuals from the non-risky and risky areas, respectively. In column 5, we interact the additive effect of the 7R allele with the dummy variable for living in the risky area. Further details on the equation used in column 5 can be found in the Methods section. Student’s *t*-test * $p<$0.10, **$p<$0.05, *** $p<$0.01. To see the correlations between controls and risk-tolerance, absent any genetic factor, see Table S.3.

## Discussion

In this paper, we found that the 7R allele of DRD4 affects risk-attitudes by an additivity effect, not a dominance effect. This is in contrast with previous research^37^ who found that heterozygotes 2R/4R had lower risk tolerance. It is unclear if this difference comes from the type of risky environment considered (background volcanic risk or risky subsidence type), or comes from the different alleles involved (2R and 4R in Indonesia, or 7R in Senegal). In addition, we did not find evidence that the 7R allele is associated with novelty seeking, as previously found^38^.

The main result of this paper is that there is no difference in the effect of the 7R allele of the DRD4 locus on risk-tolerance, depending on the risk to which the participants were exposed (Table 2, column 5). Two reasons could be advanced, then, to explain why the DRD4 locus did not reach statistical significance in the GWAS analysis on risk-taking behavior^4^. First, the measure of risk-tolerance in this study differs from the GWAS study: while ours is a behavioral measure, theirs is based on a survey question. Second, as GWAS can usually only test for the effects of Single Nucleotide Polymorphisms (SNPs), it did not directly measure the one due to the DRD4 VNTR. However, as the 7R allele is under strong positive selection, SNPs with a high linkage disequilibrium to this VNTR should have been detected^14,15^.

The sample would satisfy conditions for a genetic adaptation to habitat^39,40^, with (1) limited migration - 68% of grandparents of the risky area were born in the same village (Table S.6), (2) strong economic benefit to live in risky area and (3) an heritable genetic trait which would help cope with risk, i.e. the 7R allele. However, we did not find evidence of genetic differentiation at the DRD4 locus relative to 29 unlinked microsatellites loci (Table S.8 and Figure S.2). Moreover, if there was genetic differentiation, it would move in the opposite direction as the risk-vulnerability hypothesis found in previous work for DRD4^37^, as the 7R allele, favoring more risk-tolerant attitudes, is more prevalent in the risky area. Altogether, our results indicate that no selection at the DRD4 locus is apparent in our sample. This does not mean that such selection is absent, as many generations of selection are required for gene frequencies to change. This dangerous fishing activity started perhaps around the 16th century^41^, thus, with 4–5 generations per century, this gives approximately 20-25 generations for which selection could have occurred, which is small. It is thus unclear if selection is acting, but during a too short period of time, or if there is currently no selection at the DRD4 locus.

Another point worth mentioning is that the observed differences between zones could also reflect the effects of occupation on risk attitudes, because of a strong correlation between the living area and the probability of being a fisherman (85% of the sample in the risky area declared their main activity as fishing, vs. 4% in the non-risky area).

There are two potential confounding factors in this study. First, our results could be driven by differences in income between fishermen and non-fishermen. Fishermen indeed are richer than non-fishermen (Table S.1). Because the proportion of missing values represented 29% of our sample (details in Supplementary Materials), imputing missing values was recommended^42^. For this purpose, we used both random forest methods^43^ and Lasso regularization for imputations. No correlation was found between income and risk-tolerance in our sample (Table S.9, column 1), although this had been previously found elsewhere^44^. After imputing the missing values with either of these methods and controlling for income, the effect of the 7R allele on risk-tolerance remained significant (Table S.9, columns 3 and 4).

A second potential confounding factor is the experience with commerce or financial activities. Prior work has suggested that market integration of a community can impact decision-making in behavioral economics paradigms^45^. It is possible that the two populations differ in this aspect, but no information is available on this point in our dataset.

Further work should focus on genetic adaptation at other loci, for instance using the loci identified in the GWAS on risk attitudes^4^. Moreover, identifying other solutions for people to cope with risk in risky environments could also be further investigated.

## Methods

A field study was conducted in the Saint-Louis region in Northern Senegal between March 2018 and March 2020. All experiments were conducted in accordance with relevant guidelines and regulations. The protocol (including genotyping) was approved by the Senegalese National Ethics Committee (*Comité National d’Ethique en Recherche en Santé*), and informed consent was obtained from all participants. Behavioral measures were made at the same time as samples were collected for genotyping, so genotypes were not established at the time of measure. Investigators were blind to the behavioral measures during the genotyping.

### Measure of risk-tolerance

    We relied on a standard measure of risk-elicitation task from the experimental economics literature^36^. Instructions were displayed in French (the official written language of the country) and enumerators were present to explain the instructions in Wolof, the vernacular language of Senegal. Participants were invited to choose a card among five. On each card, two amounts were displayed, with an associated color (red or black) and the corresponding amount in coins of XOF 100, in order to have a more visual representation. At the end of the experiment, one ball was randomly drawn by a local child and gains were calculated. The cards ranged from completely risk-free (400 XOF for both balls) to extremely unequal (0 XOF if Red, 1200 XOF if Black). At each new card, the risk is increased, but so is the average amount won. Participants performed the task once. Cards used are displayed in Figure S.3.

### Genotyping

    DRD4 genotyping was done as described in^37^. In short, DNA was collected on FTA paper and extracted according to the manufacturer’s instruction. 506 and 211 samples from the risky and non risky area respectively were of sufficient quality to allow amplification with the appropriate primers. Relevant allele was estimated by the size of the PCR product on a 2% agarose gel.

The variable *7R: additive effect* is equal to the number of 7R alleles for the individual, while the variable *7R: dominance effect* is a dummy variable equal to 1 when the individual possesses only one allele 7R.

Microsatellite genotyping was based on high-throughput sequencing technology (SSRseq). 190 samples of each area were picked up randomly with the sample() function in R. 30 microsatellite tests were designed according to a streamlined SSRseq development workflow described in^46^, of which 29 gave differentiation information (one had only one allele for all individuals). The genomic localization of the 29 microsatellites and their corresponding $F_{ST}$ between the 2 populations are presented in Table S.8. Details on the design and analysis are in supplementary materials.

### Population genetics

    DRD4 locus was tested for conformity with Hardy-Weinberg (HW) equilibrium using the exact probability test^47^. Deviations from HW equilibrium were measured using the $F_{IS}$ estimator^48^. DRD4 and microsatellite loci genotypic differentiation between populations was tested for by calculating an unbiased estimate of the P-value of a log-likelihood (G) based exact test^49^, a global test over loci was calculated using Fisher’s method. Population differentiation was measured using the $F_{ST}$ estimator^48^. Calculations were performed using Genepop R package (V. 1.1.7), based on^50^.

### Statistical analysis

    In the tables and figures, the significance levels are calculated using Student’s *t*-test, comparing the ratio of the effect size to the standard error. For column 5 of Table 2, the estimated equation is the following, where *i* denotes the individual.

1 $$\begin{aligned} risk\, tolerance_i= & {} \beta _0 + \beta _1 7R:additive\, effect_i + \beta _2 7R:dominance\, effect_i\nonumber \\{} & {} + \beta _3 Age_i + \beta _4 Education_i + \beta _5 Risky\, area_i \nonumber \\{} & {} + \beta _6 7R:additive\, effect_i \times Risky\, area_i + \epsilon _i \end{aligned}$$

A significant positive (resp. negative) coefficient for the interaction term ($\beta _6$) would indicate that the additive effect of the 7R allele on risk-tolerance is stronger (resp. weaker) in the risky area.

A power analysis was calculated before the genetic analysis was performed. Using Dagnelie’s formula, with a 520 samples, an allelic difference of 0.1 could be detected with $p=0.05$ with a power of $1-\beta = 0.90$.

## Supplementary Information

Supplementary Information.

## Data Availability

The data used for this paper are available on the repository of the American Economic Association, under the identifier “openicpsr-179321”, and can be accessed using the following link (login necessary). The sequencing data are registered on the BioProject data base, under the identifier ID PRJNA879442, and are accessible using the following link (embargo until 2023-10-05).
