# Supplementary material for: The effect of the 7R allele at the DRD4 locus on risk tolerance is independent of background risk in Senegalese fishermen
Source: Sci Rep. 2023 Jan 12;13:622. doi: 10.1038/s41598-022-27002-3 (PMC9837182; doi:10.1038/s41598-022-27002-3)
Supplement: Supplementary file 1 — Supplementary Information. [file 41598_2022_27002_MOESM1_ESM.pdf]

The effect of the 7R allele at the DRD4 locus on risk tolerance is independent of background risk in Senegalese fishermen
Supplementary Materials

Gwen-Jiro Clochard
Aby Mbengue
Clément Mettling
Birane Diouf
Charlotte Faurie
Omar Sene
Emilie Chancerel
Erwan Guichoux
Guillaume Hollard
Michel Raymond
Marc Willinger

December 2022

Supplementary Materials

Differences in income between fishermen and non-fishermen

Table S.1: Differences in income for fishermen

	(1)<br>Income level
Fisherman	0.958***
	(0.138)
Constant	2.211***
	(0.109)
R^2	0.073
No. obs	616

The outcome variable is the declared income level. Standard errors between parentheses. Both risky and non-risky areas were combined. Student's t-test * p<0.10, ** p<0.05, *** p<0.01.

Descriptive statistics

Table S.2: Descriptive statistics

Variable	Mean	Std. Dev.	Min.	Max.	N
Panel A. Combined					
Age	28.978	12.462	18	92	860
Education	3.023	2.990	0	9	835
Income	2.808	1.718	1	6	616
Fisherman	0.609	0.488	0	1	864
Panel B. Risky area only					
Age	28.267	10.881	18	92	600
Education	2.404	2.717	0	9	581
Income	3.080	1.836	1	6	425
Fisherman	0.857	0.350	0	1	601
Panel C. Non-risky area only					
Age	30.619	15.408	18	71	260
Education	4.437	3.107	0	9	254
Income	2.204	1.225	1	6	191
Fisherman	0.042	0.201	0	1	263

Descriptive characteristics of the sample, for the combined sample, and disaggregated by zone. Age is calculated in years, education in years of formal schooling. Income is a categorical variable with the declared weekly income (1: < XOF 15 000, 2: 16 000 to 35 000, 3: 36 000 to 45 000, 4: 46 000 to 55 000, 5: 56 000 to 75 000, 6: > 76 000).

Differences in behavior between zones and genotypes

Table S.3: Differences between zones in risk-tolerance

	(1) Without controls	(2) With controls
Risky area	-0.099***	-0.105***
	(0.027)	(0.029)
Age		-0.002
		(0.001)
Education		0.000
		(0.005)
Constant	0.544***	0.591***
	(0.022)	(0.048)
R^2	0.016	0.019
No. obs	860	833

The outcome variable is risk-tolerance. A higher level of risk-tolerance indicates the choice of a riskier lottery by participants in the lottery choice task. Standard errors in parentheses. The coefficients are the results of Ordinary Least Square (OLS) estimations. Student's t-test * p<0.10, ** p<0.05, *** p<0.01.

Figure S.1: Distribution of risk-tolerance by genotype by zone

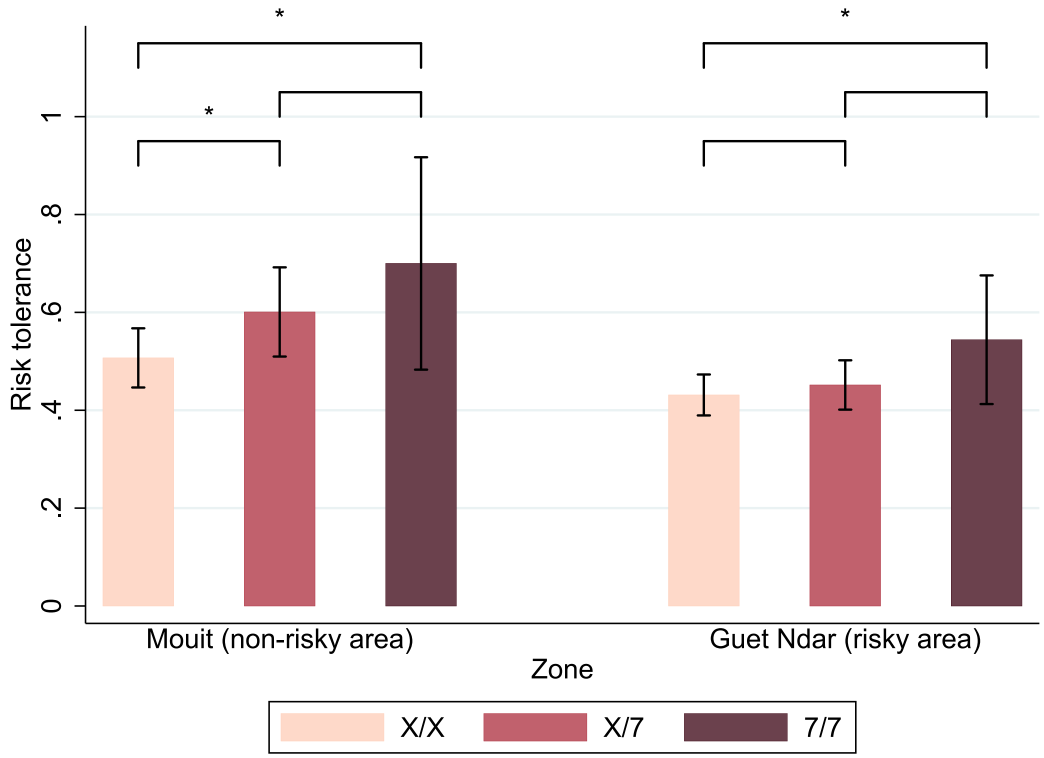

Note: The higher the risk-tolerance variable, the riskier the choice of participants. X/X, X/7 and 7/7 represent genotypes, with all alleles not 7R combined into the X allele. Segments represent 95% confidence intervals. * $p < 0.1$, ** $p < 0.05$, *** $p < 0.01$.

Table S.4: Effects of control variables on risk-attitudes

	Combined sample (1)	Non-risky area only (2)	Risky area only (3)
Age	−0.002	0.002	−0.003**
	(0.001)	(0.002)	(0.001)
Education	0.0002	0.013	−0.004
	(0.005)	(0.009)	(0.005)
Risky area	−0.105***		
	(0.029)		
Constant	0.591***	0.435***	0.543***
	(0.048)	(0.083)	(0.044)
Observations	833	252	581
R^2	0.019	0.010	0.010

Note: The outcome variable is risk-tolerance. A higher level of risk-tolerance indicates the choice of a riskier lottery by participants in the lottery choice task. Standard errors in parentheses. The coefficients are the results of Ordinary Least Square (OLS) estimations. In column 1, the sample is pooled (non-risky and risky areas). In columns 2 and 3, the sample is restricted to individuals from the non-risky and risky areas, respectively. Student's t-test * p<0.10, ** p<0.05, *** p<0.01.

Novelty seeking

Task We asked participants to choose one of two options. The first option was to drink one glass of a well-known and well recognizable soda. The second option was to drink two cups of a "novel" soda, which was prepared by the research team and was a mixture of a famous brand of soda and fruit juice. The variable $Novelty$ is defined as a dummy variable equal to 1 if the participant chose the novel soda.

Table S.5: Differences between genotypes in novelty-seeking behavior

	Combined sample<br>(1)
7R: additive effect	0.048
	(0.121)
7R: dominance effect	−0.007
	(0.157)
Age	0.013***
	(0.004)
Education	0.036*
	(0.021)
Risky area	−0.378***
	(0.132)
Constant	−1.178***
	(0.222)
Observations	661

Note: The outcome variable is novelty seeking. The variable for novelty seeking is a choice between two beverages, one being a standard easily recognizable, and the other being a new soda, unknown to participants. Standard errors in parentheses. The coefficients are the results of a Probit estimations. The sample is pooled (non-risky and risky areas). Student's t-test * p<0.10, ** p<0.05, *** p<0.01.

Analysis of genetic differentiation between zones

Table S.6: Percentage of ancestors born in the same village as participants

	(1) Risky area	(2) Non-risky area	(3) Total
Participants	81	67	77
Total parents	*72*	*58*	*68*
Mother	71	67	66
Father	73	49	70
Total grandparents	*68*	*50*	*62*
Maternal grandfather	67	42	59
Maternal grandmother	69	51	63
Paternal grandfather	67	58	64
Paternal grandmother	68	49	62

Figure S.2: Distribution of genotypic differentiation between the risky and non-risky areas

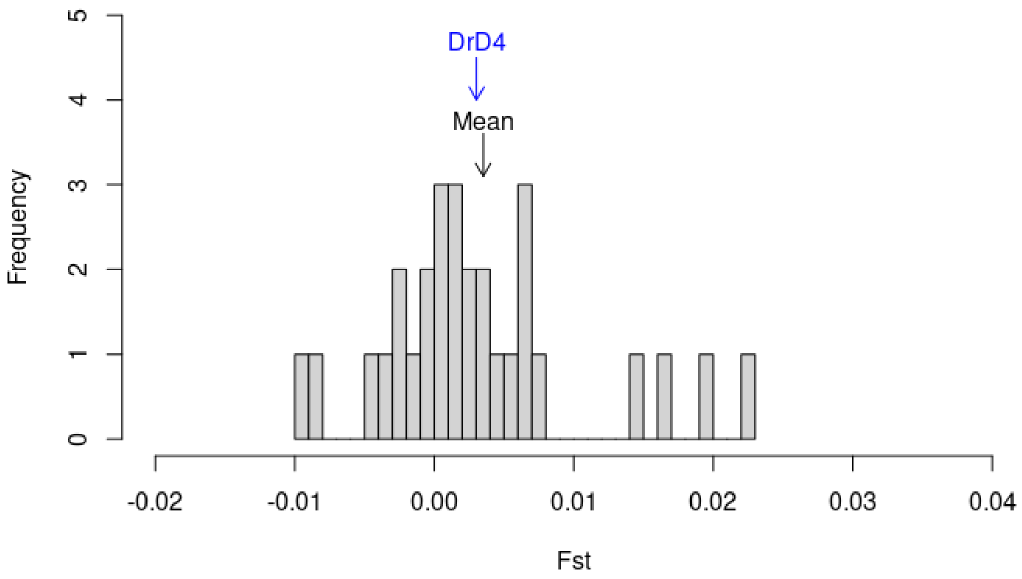

F_{ST}-statistics for genotypic differentiation between the two areas, for the 29 microsatellite loci. The mean F_{ST} is indicated by an arrow, and the F_{ST} for DRD4 is indicated by another arrows.

Micro-satellite analysis

Microsatellite development MicroSatellite DataBase (MSDB) v3 (Avvaru et al., 2020) was used to retrieve sequences containing SSR that are relatively well distributed across all chromosomes and located on intergenic regions. The motifs that have been retained were: AC and AG with a minimum of 9 repetitions; ACT, AGC and ATC with a minimum of 8 repetitions; ACTC, ACAG, ACTG, AGCT and ATGC with a minimum of 6 repetitions. The recovered sequences included the motif as well as the 200 bp upstream and downstream of this motif. A total of 5340 sequences were kept and analysed with QDD version 3.1.2. QDD primer design parameters were optimised for multiplex PCR as mentioned in Lepais et al. (2020): amplicon length between 100 and 180 bp, primer size between 21 and 26 bp, primer Tm between 60 and 75 °C, Tm difference between primers $\leq$ 10 °C and GC percent between 40 and 60. A total of 60 primer pairs were recovered and tagged at the 5-end with universal Illumina adapter overhang sequences: TCGTCGGCAGCGTCAGATGTGTATAAGAGACAG for forward primers and GTCTCGTGGGCTCGGAGATGTGTATAAGAGACAG for reverse primers, see Table S.8 for SSR localization on human chromosome and Table S.7 for primer list.

Amplification of all primers pairs was realised independently on a DNA pool of 10 individuals. The PCR was prepared in a final volume of 10 μL containing 2 μL of 5X Hot FIREpol Blend Master Mix (Solis Biodyne, Tartu, Estonia), 1 μL of 2μM primer pairs, 1 μL of the DNA pool (14 ng/μL) and 6 μL of PCR-grade water. The PCR conditions consisted of an initial denaturation at 95°C for 15 min followed by 35 cycles of denaturation at 95°C for 20 s, annealing at 59°C for 60 s, extension at 72°C for 30 s, and a final extension step at 72°C for 10 min. The amplification of each primer pair was assessed on 3% agarose gel electrophoresis. Primer pairs that did not amplify or showed an non-specific profile were removed from further analyses.

Genotyping analyses Genotyping analyses included 95 DNA samples in replicates (including a negative control) to select loci that produced repeatable genotypes for the final genotypic data set.

PCR multiplexing of all selected markers was performed in a volume of 10μL using 2 μL of 5X HOT FIREPol MultiPlex Mix (Solis Biodyne), 1 μL of multiplex primer mix (0.5 μM), 5 μL of DNA (3 ng/μL) and 2 μL of PCR-grade water. The PCR conditions consisted of an initial denaturation at 95°C for 12 min followed by 35 cycles of denaturation at 95°C for 30 sec, annealing at 59°C for 180 sec, extension at 72°C for 30 sec, and a final extension step at 72°C for 10 min. PCR products were checked by gel electrophoresis in 3% agarose.

The amplicons were amplified in a second step in order to attach Illumina adapters and dual indexes (8bp unique sequences) to each side of the amplicons.

This PCR was performed in a final volume of 20 μL using 5X HOT FIREPol MultiPlex Mix, 5 μL of amplicon and 0.5 μM of each of the forward and reverse adapters. The PCR conditions consisted in an initial denaturation at 95°C for 12 min followed by 15 cycles of denaturation at 95°C for 30 s, annealing at 59°C for 90 s, extension at 72°C for 30 s, and a final extension step at 72°C for 10 min. The second PCR-barcoded amplicons were pooled in a single tube, purified with 1.8X Agencourt AMPure XP beads (Beckman Coulter, Brea, USA) and analysed on a on a Tapestation 4200 (Agilent, Santa Clara, USA). The final pool was quantified using QIAseq Library Quant Assay kit (Qiagen, Hilden, Germany) with a Roche LightCycler 480 quantitative PCR (Roche, Penzberg, Germany) and sequenced on an iSeq 100 system (Illumina, San Diego, USA) with a 2x150 bp kit.

The bioinformatic pipeline embedding FDSTools (Hoogenboom et al., 2017) and described in Lepais et al. (2020) was used to compare blind-repeated genotyping to estimate the genotyping error rate and to extract locus and allele information.

Results Of the 60 starting markers, only 54 gave a specific amplification of which 24 were discarded due to genotyping error of more than 5% or a high missing rate due to poor DNA recovery. Of the 30 remaining, 18 had a dinucleotide motif and 12 a trinucleotide motif and were distributed on 17 chromosomes in total (Table S.8). A total of 186 alleles differing in sequence (mean: 12 alleles per loci) and 159 alleles differing in size (mean 10.25 alleles per loci) were observed and one locus was identified as monomorphic.

Table S.7: Primer sequence for micro-satellites

Locus	Sequence
SSRSEQ_002F	TCGTCGGCAGCGTCAGATGTGTATAAGAGACAGGGGATGTTAATAGCACCTGTTTCACG
SSRSEQ_005F	TCGTCGGCAGCGTCAGATGTGTATAAGAGACAGATGCTCACACCATTGCACTCCAGCG
SSRSEQ_007F	TCGTCGGCAGCGTCAGATGTGTATAAGAGACAGGCTTCCAAGCTGGGAACGCAGTGTC
SSRSEQ_010F	TCGTCGGCAGCGTCAGATGTGTATAAGAGACAGCCTTGCAAGGTAGGGTGGCGAGCAT
SSRSEQ_011F	TCGTCGGCAGCGTCAGATGTGTATAAGAGACAGGGATGGCTTGGGCAATACTCTTGGT
SSRSEQ_013F	TCGTCGGCAGCGTCAGATGTGTATAAGAGACAGCCCAAAGCTGAATAAGTCTGTCCAC
SSRSEQ_014F	TCGTCGGCAGCGTCAGATGTGTATAAGAGACAGAACCAAAGTTGATTGCAGGTGGCTGC
SSRSEQ_017F	TCGTCGGCAGCGTCAGATGTGTATAAGAGACAGTTGTGGGTGTGCATGTGAGCAGGTG
SSRSEQ_018F	TCGTCGGCAGCGTCAGATGTGTATAAGAGACAGACCATTCAGAAATAGCAGCCACTGT
SSRSEQ_021F	TCGTCGGCAGCGTCAGATGTGTATAAGAGACAGTCAGCAGGACAGTCTGGAGAGGAGG
SSRSEQ_023F	TCGTCGGCAGCGTCAGATGTGTATAAGAGACAGCCCGGTCTGTGGCTAGCTTTGATGG
SSRSEQ_025F	TCGTCGGCAGCGTCAGATGTGTATAAGAGACAGTTGCATGATCCAAAGAAGGTGGCCT
SSRSEQ_027F	TCGTCGGCAGCGTCAGATGTGTATAAGAGACAGCAAATGACTCCGCAGCTGCTGGCAC
SSRSEQ_028F	TCGTCGGCAGCGTCAGATGTGTATAAGAGACAGTCACCGGAATCATGTGGGCATCAGA
SSRSEQ_029F	TCGTCGGCAGCGTCAGATGTGTATAAGAGACAGACAGGTGAGGAAACTGAGGCTGAAA
SSRSEQ_033F	TCGTCGGCAGCGTCAGATGTGTATAAGAGACAGGCCTATCCATCCAGCTACCAACCCT
SSRSEQ_034F	TCGTCGGCAGCGTCAGATGTGTATAAGAGACAGTGGTTTCCTCTTCCCTAACCTCACT
SSRSEQ_035F	TCGTCGGCAGCGTCAGATGTGTATAAGAGACAGGCCTTTCTAGAGATGAAGACAGGGT
SSRSEQ_039F	TCGTCGGCAGCGTCAGATGTGTATAAGAGACAGTGGGAAAGTCCAGTTCCTGCTCCGT
SSRSEQ_040F	TCGTCGGCAGCGTCAGATGTGTATAAGAGACAGACAGTTCTGGAAGCTGCAAAGTCCA
SSRSEQ_041F	TCGTCGGCAGCGTCAGATGTGTATAAGAGACAGAGACCGAGTGGCCTTCAGCAAGTCA
SSRSEQ_044F	TCGTCGGCAGCGTCAGATGTGTATAAGAGACAGAGCAGGAACTTGTGCACCACATGGC
SSRSEQ_047F	TCGTCGGCAGCGTCAGATGTGTATAAGAGACAGTGGCTACCCATATTCTCAGCCCTGT
SSRSEQ_048F	TCGTCGGCAGCGTCAGATGTGTATAAGAGACAGGCAGGAGCCAGGTTACAGAGGGTTT
SSRSEQ_049F	TCGTCGGCAGCGTCAGATGTGTATAAGAGACAGTGGCACACCTACTAAGTCCCAGGCA
SSRSEQ_057F	TCGTCGGCAGCGTCAGATGTGTATAAGAGACAGTTGCCACCCTAGAGACAGGCTCAGC
SSRSEQ_058F	TCGTCGGCAGCGTCAGATGTGTATAAGAGACAGCCAGCTTGCTGACTGTAGATCTCGGG
SSRSEQ_059F	TCGTCGGCAGCGTCAGATGTGTATAAGAGACAGACATGAGAGCCGAGTATGTACTGGA
SSRSEQ_060F	TCGTCGGCAGCGTCAGATGTGTATAAGAGACAGGGCAGGTCAGGAGGGTTCTGTCCTT
SSRSEQ_002R	GTCTCGTGGGCTCGGAGATGTGTATAAGAGACAGATGCCAGGAATTGTTCTAAGCATGT
SSRSEQ_005R	GTCTCGTGGGCTCGGAGATGTGTATAAGAGACAGAGGAGGCGAAGGGACCCTGGTAGAT
SSRSEQ_007R	GTCTCGTGGGCTCGGAGATGTGTATAAGAGACAGGGGATACAGGAGGACTTCAGAGTCA
SSRSEQ_010R	GTCTCGTGGGCTCGGAGATGTGTATAAGAGACAGCCATTTGCCATGGTCCCAGGAACTC
SSRSEQ_011R	GTCTCGTGGGCTCGGAGATGTGTATAAGAGACAGGCAGAAGGCGAAGTGGGCATAAGCG
SSRSEQ_013R	GTCTCGTGGGCTCGGAGATGTGTATAAGAGACAGTGGTGATGCCACTCATGAGGGAGCT
SSRSEQ_014R	GTCTCGTGGGCTCGGAGATGTGTATAAGAGACAGGGCTGCAGTTGTTTCACACCACAGCT
SSRSEQ_017R	GTCTCGTGGGCTCGGAGATGTGTATAAGAGACAGACTCCCTTCAACTCCAAGCGTGCCT
SSRSEQ_018R	GTCTCGTGGGCTCGGAGATGTGTATAAGAGACAGGTGAGGAATAGAAGCTGGACCCTTGT
SSRSEQ_021R	GTCTCGTGGGCTCGGAGATGTGTATAAGAGACAGCTGTGGGTTTGCAGCATGAGACGCA
SSRSEQ_023R	GTCTCGTGGGCTCGGAGATGTGTATAAGAGACAGGACACCAGAACACAAAGGGCACACC
SSRSEQ_025R	GTCTCGTGGGCTCGGAGATGTGTATAAGAGACAGTGGCTGGCATTAATTATGTTCCAGA
SSRSEQ_027R	GTCTCGTGGGCTCGGAGATGTGTATAAGAGACAGCGTTTGCCACCAGGACTGTCCCAAC
SSRSEQ_028R	GTCTCGTGGGCTCGGAGATGTGTATAAGAGACAGACAGTCTCCTCCAGAAATGCGGATT
SSRSEQ_029R	GTCTCGTGGGCTCGGAGATGTGTATAAGAGACAGAAAGACCATCTTCTCCCTCTGCTTT
SSRSEQ_033R	GTCTCGTGGGCTCGGAGATGTGTATAAGAGACAGCACCCAGTGCATGTTTACCCGAGGA
SSRSEQ_034R	GTCTCGTGGGCTCGGAGATGTGTATAAGAGACAGATGTTGTCACATAGGCGTGCAGGCA
SSRSEQ_035R	GTCTCGTGGGCTCGGAGATGTGTATAAGAGACAGGCACCTAGCAAGCACTTAACAAGCA
SSRSEQ_039R	GTCTCGTGGGCTCGGAGATGTGTATAAGAGACAGCCAGGGATGGCCTTGCACCAACATA
SSRSEQ_040R	GTCTCGTGGGCTCGGAGATGTGTATAAGAGACAGAGGACTATGGCCTTATAAGAAGAGGA
SSRSEQ_041R	GTCTCGTGGGCTCGGAGATGTGTATAAGAGACAGTGTCACTGCCATCAACCCTTTCGTT
SSRSEQ_044R	GTCTCGTGGGCTCGGAGATGTGTATAAGAGACAGGCTTGGGCCATCTTCTTGGTGATAA
SSRSEQ_047R	GTCTCGTGGGCTCGGAGATGTGTATAAGAGACAGAAAGGTACAATAGGTGGTCCAGGTA
SSRSEQ_048R	GTCTCGTGGGCTCGGAGATGTGTATAAGAGACAGTCCACCATCCTCTTTCCCTAGCTGGT
SSRSEQ_049R	GTCTCGTGGGCTCGGAGATGTGTATAAGAGACAGTAGGTGAACTGCTTAGCACATACGT
SSRSEQ_057R	GTCTCGTGGGCTCGGAGATGTGTATAAGAGACAGACCACTAGCAGGGTAGCTGGAGCAT
SSRSEQ_058R	GTCTCGTGGGCTCGGAGATGTGTATAAGAGACAGCGTGAGTCAATGAGTCATTTGAGGA
SSRSEQ_059R	GTCTCGTGGGCTCGGAGATGTGTATAAGAGACAGTGGTCCCAGCTATACTCCATACTTG
SSRSEQ_060R	GTCTCGTGGGCTCGGAGATGTGTATAAGAGACAGCCAAGCTGGTGCCTTGAATACTGCA

Table S.8: Genetic differentiation between the non-risky and risky areas on micro-satellites.

**Locus**	**Motif**	**Chromosome: position**	**N alleles Sequence**	**N allele Size**	**Allelic Error**	F_{ST}
SSRSEQ_002	AC	chr1:25140881-25140899	12	8	2.32%	0.0053
SSRSEQ_027	AC	chr2:9753457-9753484	7	7	0%	-0.0081
SSRSEQ_028	CAG	chr2:16239536-16239584	17	12	0.76%	0.0010
SSRSEQ_029	AGC	chr2:44975447-44975477	9	9	3.12%	0.0067
SSRSEQ_039	TG	chr3:13815554-13815575	8	8	4.00%	0.0069
SSRSEQ_040	AG	chr3:28213984-28214005	3	3	0%	-0.0015
SSRSEQ_041	GAT	chr4:1467533-1467565	5	5	0.60%	0.0067
SSRSEQ_044	GA	chr5:12484633-12484657	8	8	0.98%	-0.0001
SSRSEQ_047	CA	chr6:12334400-12334425	6	6	0.64%	-0.0024
SSRSEQ_048	AGC	chr6:21033972-21033999	7	5	1.68%	0.0199
SSRSEQ_049	CAT	chr6:34463481-34463513	4	4	0%	-0.0048
SSRSEQ_057	CAT	chr8:37410868-37410898	7	4	0.62%	-0.0023
SSRSEQ_058	TG	chr9:4357561-4357589	12	11	4.00%	0.0033
SSRSEQ_059	AC	chr9:10942052-10942074	3	3	0.76%	-0.0010
SSRSEQ_060	TC	chr9:23340635-23340654	5	4	0%	0.0017
SSRSEQ_005	GA	chr10:7553026-7553045	6	6	0.78%	0.0012
SSRSEQ_007	TC	chr11:2009564-2009584	2	2	0%	0.0007
SSRSEQ_010	GAT	chr12:607351-607380	2	2	0%	0.0041
SSRSEQ_011	TC	chr12:41680046-41680064	3	3	0.72%	-0.0033
SSRSEQ_013	ATG	chr13:24648458-24648485	7	5	2.87%	0.0027
SSRSEQ_014	AGC	chr13:67656823-67656870	11	11	0%	0.0079
SSRSEQ_017	GT	chr16:5398567-5398589	8	5	1.92%	0.0226
SSRSEQ_018	AC	chr16:12462593-12462611	4	4	0%	0.0150
SSRSEQ_021	CTG	chr17:49684195-49684223	3	3	0%	0.0008
SSRSEQ_023	GT	chr19:6517969-6517988	2	2	0%	0.0165
SSRSEQ_025	GA	chr19:28667178-28667198	4	4	0.73%	0.0022
SSRSEQ_033	ATC	chr20:56130369-56130397	3	3	0%	-0.0094
SSRSEQ_034	TG	chr21:5520201-5520225	12	6	4.54%	0.0040
SSRSEQ_035	CTA	chr21:15100302-15100333	5	5	0%	0.0001
Combined						0.0035

The measure of differentiation F_{ST} refers to the estimate from Weir and Cockerham (1984).

Measure of risk-tolerance

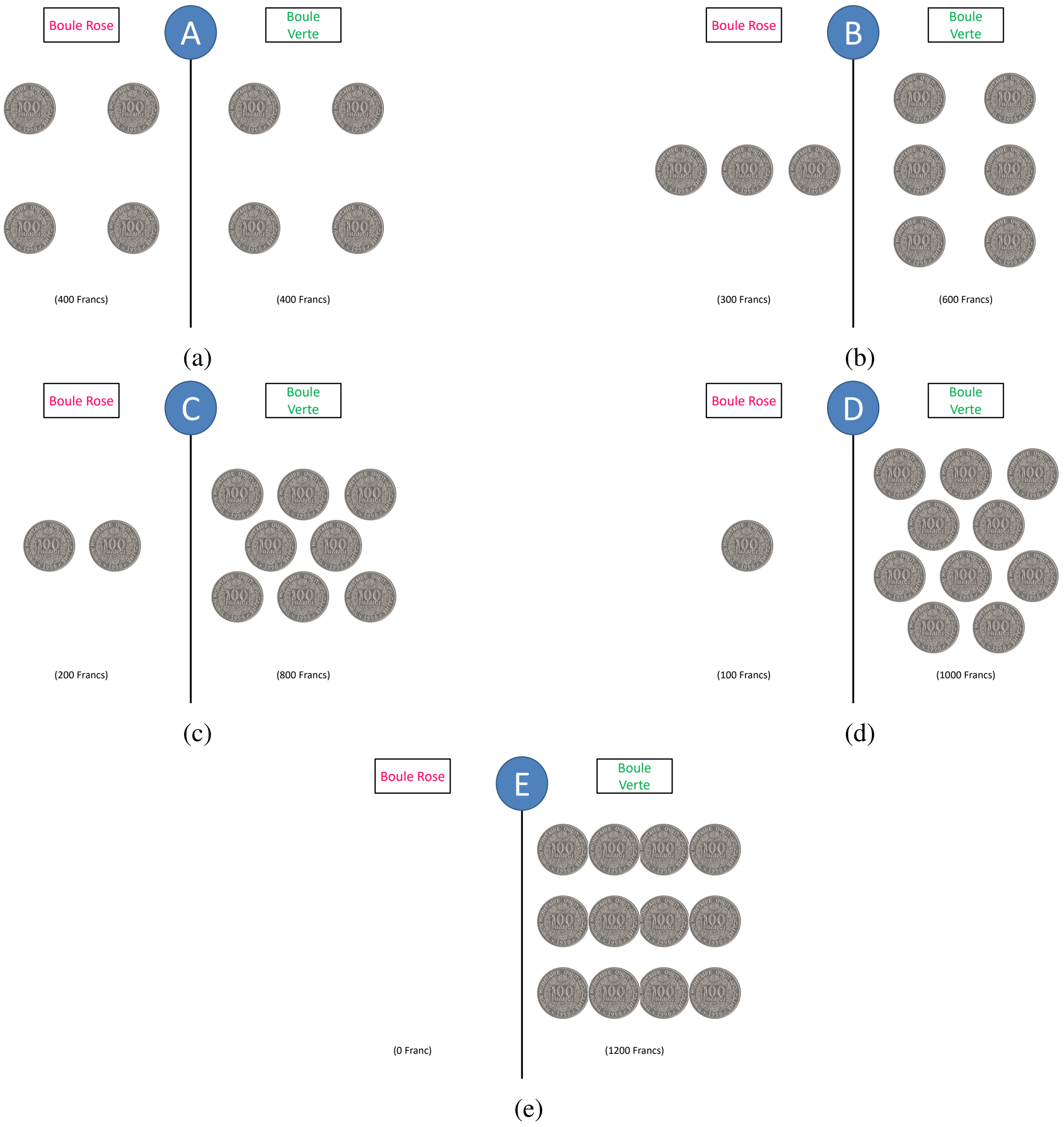

Figure S.3: Cards displayed in the risk-tolerance elicitation task.

Note: Participants were asked to choose a card among these five. The result is a variable *Choice* from 1 to 5. The variable *Risk-tolerance* was then computed using the following formula: $Risk - tolerance = \frac{Choice-1}{4}$. The *Risk-tolerance* variable is therefore a variable ranging from 0 (the participant chose the safest option, i.e. the card A) to 1 (the participant chose the riskiest option, i.e. the card E).

Participants performed the task only once. Binswanger (1980) found no evidence that learning played a role when the task is repeated, therefore the single trial set up has become standard practice in experimental economics (see for instance Dave et al. (2010) or Holzmeister and Stefan (2021) for lab experiments, and Barr and Genicot (2008) or Strobl and Wunsch (2021) for field experiments).

Income as a confound

Method One potential confound for our results could be that the effect of the 7R allele on risk-tolerance is confounded by an effect of income on risk-attitudes. To test for this, we performed the same regression as in our main specification, adding income as a control.

However, a large fraction of our sample (29%) answered “I do not know” when asked about their income, and their answers were therefor noted as missing values. To cope for this, we used two methods for imputation. First, a Lasso estimation using all other information available in the data set (other than genotype and risk-attitudes). Second, we used a random forest algorithm (Stekhoven and Buehlmann, 2012) using, again, existing other characteristics. Both methods enabled us to create a variable called “Predicted income”, equal to the value when reported, and equal to the imputed value when missing.

Results Results are presented in Table S.9. First, we can see that income is not correlated with risk-tolerance in any specification.

Second, when controlling for income in addition to age, education and living area, the effect of the 7R allele on risk-tolerance is no longer significant. However, the much smaller sample could be a reason to worry, which is the reason why we imputed the value of income for missing values.

Third and most importantly, when predicting the income for missing values using Machine Learning algorithms, we find similar results to the main specification, i.e. the 7R allele has an additive effect, no dominance effect, and there is no significant interaction effects between the living area and the effect of the 7R allele. The magnitude of the effects of the 7R allele is unaffected by the addition of income as a control.

Table S.9: Effect of income and the 7R allele on risk-attitudes, combined sample

	(1) Effect of income	(2) Effect of 7R with income as control	(3) Effect of 7R with income imputed from Lasso	(4) Effect of 7R with income imputed from Random Forest
7R: additive effect		0.097*	0.107**	0.109**
		(0.052)	(0.045)	(0.042)
7R: dominance effect		0.016	−0.024	−0.029
		(0.045)	(0.037)	(0.036)
Age	−0.001	−0.002	−0.002**	−0.002**
	(0.001)	(0.001)	(0.001)	(0.001)
Education	0.002	−0.004	−0.004	−0.004
	(0.005)	(0.006)	(0.005)	(0.005)
Risky area	−0.134***	−0.110**	−0.094**	−0.088**
	(0.035)	(0.046)	(0.039)	(0.038)
Income	0.005	−0.001		
	(0.009)	(0.010)		
Predicted income $Lasso$			0.001	
			(0.010)	
Predicted income $RandomForest$				−0.003
				(0.009)
Risky area × 7R: additive effect		−0.085	−0.062	−0.058
		(0.058)	(0.049)	(0.047)
Constant	0.574***	0.597***	0.595***	0.600***
	(0.060)	(0.068)	(0.058)	(0.057)
Observations	607	501	699	721
R^2	0.029	0.039	0.033	0.034

The outcome variable is risk-tolerance. A higher level of risk-tolerance indicates the choice of a riskier lottery by participants in the lottery choice task. Standard errors in parentheses. The coefficients are the results of Ordinary Least Square (OLS) estimations. The "Predicted income $Lasso$" variable is constructed using a Lasso estimation, using all other variables available (except risk attitudes and genotypes). The "Predicted income $Forest$" variable is constructed using a random forest algorithm (Stekhoven and Buehlmann, 2012). Student's t-test * p<0.10, ** p<0.05, *** p<0.01.

References

Avvaru, A. K., D. Sharma, A. Verma, R. K. Mishra, and D. T. Sowpati (2020). Msdb: a comprehensive, annotated database of microsatellites. *Nucleic acids research 48*(D1), D155–D159.

Barr, A. and G. Genicot (2008). Risk sharing, commitment, and information: an experimental analysis. *Journal of the European Economic Association 6*(6), 1151–1185.

Binswanger, H. P. (1980). Attitudes toward risk: Experimental measurement in rural india. *American Journal of Agricultural Economics 62*(3), 395–407.

Dave, C., C. C. Eckel, C. A. Johnson, and C. Rojas (2010). Eliciting risk preferences: When is simple better? *Journal of Risk and Uncertainty 41*(3), 219–243.

Holzmeister, F. and M. Stefan (2021). The risk elicitation puzzle revisited: Across-methods (in) consistency? *Experimental Economics 24*(2), 593–616.

Hoogenboom, J., K. J. van der Gaag, R. H. de Leeuw, T. Sijen, P. de Knijff, and J. F. Laros (2017). Fdstools: A software package for analysis of massively parallel sequencing data with the ability to recognise and correct str stutter and other pcr or sequencing noise. *Forensic Science International: Genetics 27*, 27–40.

Lepais, O., E. Chancerel, C. Boury, F. Salin, A. Manicki, L. Taillebois, C. Dutech, A. Aissi, C. F. Bacles, F. Daverat, et al. (2020). Fast sequence-based microsatellite genotyping development workflow. *PeerJ 8*, e9085.

Stekhoven, D. J. and P. Buehlmann (2012). Missforest - non-parametric missing value imputation for mixed-type data. *Bioinformatics 28*(1), 112–118.

Strobl, R. and C. Wunsch (2021). Risky choices and solidarity: disentangling different behavioural channels. *Experimental Economics 24*(4), 1185–1214.

Weir, B. S. and C. C. Cockerham (1984). Estimating f-statistics for the analysis of population structure. *evolution 38*(6), 1358–1370.

Authors' affiliations

G-J Clochard: University of Chicago and UCEMAs Joint Initiative for Latin American Experimental Economics - Ecole polytechnique - CREST (CNRS-UMR 9194), Palaiseau, France

A. Mbengue: Université Gaston Berger, Saint-Louis, Sénégal

C. Mettling: ISEM Univ Montpellier, CNRS, IRD, Montpellier, France - IGH Univ Montpellier, CNRS, Montpellier, France

B. Diouf: Université Gaston Berger, Saint-Louis, Sénégal

C. Faurie: ISEM Univ Montpellier, CNRS, IRD, Montpellier, France

O. Sene: Université Alioune Diop, Bambey, Sénégal

E. Chancerel: Univ. Bordeaux, INRAE, BIOGECO, Plateforme Genome Transcriptome de Bordeaux, F-33610 Cestas, France

E. Guichoux: Univ. Bordeaux, INRAE, BIOGECO, Plateforme Genome Transcriptome de Bordeaux, F-33610 Cestas, France

G. Hollard: Ecole polytechnique - CREST (CNRS-UMR 9194), Palaiseau, France - CNRS

M. Raymond: ISEM Univ Montpellier, CNRS, IRD, Montpellier, France

M. Willinger: CEE-M Univ Montpellier, CNRS, INRAE, Institut Agro, Montpellier, France
